# The Impact of the COVID-19 Pandemic on the Brazilian Immigrant Community in the U.S: Results from a Qualitative Study

**DOI:** 10.3390/ijerph18073355

**Published:** 2021-03-24

**Authors:** Leticia Priebe Rocha, Raviv Rose, Annmarie Hoch, Cristiane Soares, Adriana Fernandes, Heloisa Galvão, Jennifer D. Allen

**Affiliations:** 1 Department of Community Health, Tufts University, 574 Boston Avenue, Medford, MA 02155, USA; leticia.rocha@tufts.edu (L.P.R.); rebecca.rose@tufts.edu (R.R.); annmarie.hoch@tufts.edu (A.H.); 2 Department of Romance Studies, Tufts University, 180 Packard Avenue, Medford, MA 02155, USA; cristiane.soares@tufts.edu; 3 Office of Immigrant Affairs, City of Somerville, 42 Cross St., Somerville, MA 02145, USA; afernandes@somervillema.gov; 4 Brazilian Women’s Group, 697 Cambridge St Suite 106, Brighton, MA 02135, USA; heloisa@verdeamarelo.org

**Keywords:** COVID-19, immigrants, Brazilian, Latino, immigrant health

## Abstract

While an increasing body of data suggests that marginalized groups have been disproportionately impacted by COVID-19, little has been published about the specific impact on Brazilian immigrants in the U.S. We conducted 15 key informant interviews, one of which included two participants (*n* = 16), with representatives from social service agencies, healthcare, and faith-based organizations serving Brazilian immigrants. Key informants were asked about the community’s experiences with COVID-19 testing and treatment, responses to CDC (Centers for Disease Control) guidelines, perceptions about the virus, and the pandemic’s impact on physical and mental health. Results suggest that COVID-19 has profoundly impacted Brazilian immigrants’ mental and physical health. Key informants perceived that community members faced higher risk of COVID-19 infection due to overcrowded living conditions and over-representation in public-facing and informal (e.g., housecleaning) jobs. They reported barriers to COVID-19-related healthcare services including language, immigration status, and fear of deportation. Brazilian cultural norms surrounding hygiene practices, social distancing, and information distribution have shaped the community’s pandemic response. The Brazilian community has faced extensive social, economic, and health ramifications due to the pandemic. While not unique to this community, pre-existing concerns about social disadvantage suggest a particular vulnerability of this population to the virus.

## 1. Introduction

COVID-19 has had a devastating global impact, extending far beyond physical health effects and altering nearly all aspects of daily life. The United States leads globally in COVID-19 confirmed cases and deaths, and there is growing evidence that the pandemic has disproportionately impacted marginalized groups, including immigrants [1,2]. Immigrants may be at especially high risk for COVID-19 infection due to overrepresentation in low-wage and public-facing jobs, inability to work from home or take sick leave, and living in households with multiple families [3]. 

As of this writing, few studies on COVID-19 among immigrant populations have been published, although there are numerous commentaries [4,5,6,7,8,9,10]. Several studies have highlighted the disproportionate effects of COVID-19 on Latino and/or immigrant groups. For example, in Massachusetts, Latinos (Brazilians included) make up 12.3% of the state’s population, yet constitute 30% of COVID-19 cases [11]. One study examined demographic characteristics associated with COVID-19 infections across Massachusetts cities/towns [2]. Researchers found that each 10% increase in the percentage of Latinos in a given city was associated with an increase of 258.2 cases per 100,000 [2]. Other factors associated with higher COVID-19 infection rates included a proportion of “foreign-born non-citizens,” average household size, and percentage of food service workers [2]. In addition to higher COVID-19 infection rates among immigrant communities, other studies have noted various pandemic related challenges among these groups such as higher rates of economic insecurity [12], lack of access to government supports and healthcare [13], and negative impacts on mental health and treatment adherence [14]. These findings indicate the importance of understanding the impacts of COVID-19 in immigrant communities.

Brazil is second only to the U.S. in terms of mortality and confirmed cases to date [15]. In the Greater Boston area, there are an estimated 350,000 Brazilian immigrants [16]. However, it is believed that only 45% of Brazilians in the U.S. are citizens, as compared to 83% among other Latino groups [16], which suggests that Census data may be inaccurate, given that non-citizens may be less likely to respond to the Census than citizens. In 2017, the average age for Brazilians was 33 years, with 74% being foreign-born and, on average, having arrived around 2004 [16]. It is estimated that 49% of Brazilians in the U.S. are female. Although there are no available data on COVID-19 infections or deaths among Brazilians in the US, Brazilian immigrants may experience a heavy toll due to the pandemic since the majority (72%) work in low-wage “service” jobs [16] and fears about immigration status may deter many from seeking services [17,18]. 

There has been substantial growth of the Brazilian immigrant population in the U.S. over the past 20 years, yet health data for Brazilians living in the U.S. is scarce [19,20]. The few studies on Brazilians in the U.S. have largely focused on occupational health [17,21], maternal feeding practices [22] and mental health [17,18]. In 2019, we completed a community health assessment among Brazilians in the Greater Boston area [23]. Since the pandemic began shortly after we completed this assessment, we saw a need to assess the impact of the COVID-19 pandemic on Brazilian immigrant health priorities and needs. This is particularly important since previously identified community health needs such as mental health, occupational health and safety, and domestic violence are likely to have shifted and/or been exacerbated by the pandemic [21,23,24]. We aim to add to the body of literature on Brazilian immigrants as well as the impact of COVID-19 on immigrant communities in the U.S.

## 2. Materials and Methods

We conducted qualitative key informant (KI) interviews with representatives in the Brazilian community across multiple community sectors to assess perceptions regarding the impact of the pandemic on Brazilians. Key informants can provide insights into community needs by the nature of their formal or informal roles. We developed a semi-structured interview guide (see Appendix A) based on our prior work [23] to examine: experiences with COVID-19 testing and treatment, responses to CDC guidelines, perceptions about the virus, and the pandemic’s impact on physical and mental health.

KIs from our previous study were invited to participate. Others were identified through snowball sampling [25]. Those who participated in our prior study had been identified through community partners and snowball sampling. Potential KIs were contacted by email and provided with informed consent information. We offered to conduct interviews in Portuguese or English, according to participant preference. Interviews were conducted by bilingual, bicultural research team members between June and July 2020. Interviews, which ranged from 45–75 min, took place via Zoom, and were recorded. Recordings were professionally transcribed and reviewed for linguistic and cultural accuracy. All study procedures were approved by the Institutional Review Board at Tufts University.

## 3. Results

### 3.1. Characteristics of Participants

Fifteen interviews were conducted with a total of 16 KIs; 1 interview included two participants who were interviewed together. KIs represented the following sectors: social services (*n* = 6), political appointees (*n* = 3), healthcare (*n* = 2), mental health services (*n* = 2), and religious organizations (*n* = 3). Most self-identified as Brazilian (15/16) and were female (11/16). All interviews were conducted in English. Half (8/16) of the KIs participated in our prior study. New participants were referred by previous participants or identified and recruited after a study team search of Brazilian organizations serving the community during the pandemic.

### 3.2. Qualitative Themes

We report major themes below based on the order of our interview guide. Below, we present supporting quotes for each major theme; additional supporting quotes can be found in Appendix B.

### 3.3. Beliefs about and Attitudes towards COVID-19

The majority of KIs (11/16) believed that COVID-19 infection was widespread in the Brazilian community due to factors such as overcrowded housing and employment in high-exposure jobs.

*“The Brazilian community is part of the communities that have been hit the most in terms of infection of black and brown communities across the state. Brazilians are no different from Hispanics; they live in housing with tons of people inside, and a lot of them do low-wage jobs and are exposed more to infection, and the lack of PPEs (Personal Protective Equipment) affected them a lot.”*

—Political appointee

KIs felt that the attitude of the Brazilian president, Jair Bolsonaro, had a major influence on perceptions about the pandemic among Brazilian immigrants. Respondents described that, at the beginning of the pandemic, many people were not seriously concerned. They reported that President Bolsonaro heavily influenced perceptions in the community by downplaying the pandemic, making statements that the virus was mild, discouraging mask wearing, and commenting on the effectiveness of treatments without factual basis.

*“We have a lot of people who followed what our president in Brazil says. And they believe this is something only for vulnerable people, people who are old, or they have another disease. And because they are young and hard workers, they are not going to be affected.”*

—Healthcare provider

Many noted that due to Brazilian immigrants’ high use of Brazilian media (e.g., radio, online newspapers, television) and social media (e.g., Facebook, WhatsApp), there was an extensive spread of misinformation (e.g., COVID-19 would not affect young people) or debunked theories about the virus (e.g., the Chinese government created it in a lab).

*“There’s a lot of misinformation and fake news that [social media groups] circulate, and then they send to each other. Most Brazilian citizens here, they do not speak or read in English, so they have to trust this information that they receive through WhatsApp, and much of this information is not very accurate.”*

—Social service provider

However, several KIs (8/16) reported that with the escalating infection rate in Brazil, perceptions about the severity of the pandemic in the U.S. shifted. As people increasingly saw friends or family members contract COVID-19, perceptions of higher severity increased.

*“When we started seeing more cases in Brazil, people started thinking that there was something serious… Almost everyone has at least one person, a relative, or a friend that was affected by the disease... I don’t think, in the beginning with the cases in the United States, that they were really concerned.”*

—Social service provider

### 3.4. Access to and Utilization of COVID-19 Testing and Treatment

Most KIs (9/16) emphasized that fears about deportation and the Department of Homeland Security’s “Public Charge Ground of Inadmissibility” was a major deterrent to COVID-19 testing for Brazilians. KIs directly raised the issue of the DHS “Public Charge” rule as a deterrent that made community members fearful of using services. This policy stipulates that immigrants’ utilization of government services (e.g., SNAP, SSI, Section 8 Housing) for more than 12 months within a 36 month period will be considered grounds for rejection of application to become lawful permanent residents [26]. Although DHS announced that COVID-19 testing and treatment is not one of the services to be considered under this rule, families remained fearful, particularly in the beginning of the pandemic.

*“What we know is that many people in the Brazilian community... are undocumented, they are usually afraid of looking for any government service because most people are afraid… to be deported. People are very reticent in looking for hospitals and police stations. So, I believe that many people may be avoiding testing because they may be afraid of looking for hospitals and these testing sites.”*

—Social service provider

Additionally, many KIs reported that COVID-19 testing centers initially required health insurance, which decreased access to testing since many Brazilians are uninsured. The provision of free COVID-19 testing through local health departments eliminated insurance as a deterrent, although additional barriers—including low English-language proficiency, the inability to take time off work, and childcare needs—compounded barriers for some. 

Furthermore, all KIs reported that Brazilians are less likely to pursue testing for medical conditions unless they experience serious symptoms, which they described as a cultural characteristic of the population. In the case of COVID-19, they emphasized that a large percentage of the Brazilian community would only seek testing if seriously ill.

*“Brazilians, it’s part of our culture, we do not go to the doctor until we are really bad, we like to medicate ourselves, right? It’s like, ‘Oh, I have the headache, I’m gonna take something. Oh, now I have this body ache, I’m gonna take this,’ so we really go to the doctor when we can’t move; that’s the reality.”*

—Political appointee

Most KIs (13/16) noted that language was a major barrier to accessing services; people were concerned about testing positive and being forced to stay in the hospital without the ability to communicate effectively with providers. As a result, many avoided testing for as long as possible.

*“The information on how to seek help really caused a delay to diagnose and get to the Brazilian community. Because a lot of them, they wouldn’t feel comfortable ever calling 911 because they don’t see 911 as a medical emergency. They think that’s police only. They faced a language barrier, and then the barrier of being afraid in the middle of an emergency that they would have to call 911. They try to ride it for as long as they could until they really had to [seek care]”*

—Political appointee

### 3.5. Responses to Mitigation Strategies

Nearly half of the KIs (7/16) believed that contact tracing would be unsuccessful among immigrant communities due to fears surrounding documentation status. They reported that community members were unlikely to pick up the phone for unknown numbers and would be unwilling to provide information regarding their location or recent contacts. Contact tracing was also complicated by language barriers, especially in the early stage of the pandemic, because contact tracers rarely spoke Portuguese and there was limited access to Portuguese translation services.

*“… there were concerns because some people supposedly are not here [undocumented]. And then, if you have to report the people you’ve been with, it exposes them, especially if you’re contacted by somebody not speaking a language. Because they wanna know who you’ve been with for the past two weeks, and…you have to report all these people that are here undocumented that don’t feel comfortable being revealed.”*

—Political appointee

A few KIs (2/16) described that culturally, Brazilians are very hygiene-oriented, therefore more frequent handwashing and sanitizing practices did not require significant adjustment to regular practice and was widely accepted.

*“In our culture, we do wash our hands a lot. It just kind of complemented what we usually do. So, it wasn’t a drastic change in habits.”*

—Mental health provider

However, most KIs (11/16) reported a high level of resistance to wearing masks and reported that people often wore their masks incorrectly. They also described community members’ disapproval of enforcement of mask-wearing when entering public spaces.

*“Masks have been a big problem… They wear the masks in the wrong way, on their chin. And they–when they want to talk, they remove the masks... when you go to Brazilian stores, we still have people who try to enter without having a mask.”*

—Healthcare provider

Twelve out of sixteen KIs also reported that social distancing was difficult for Brazilians, citing that Brazilian culture is very social, and that physical touch (e.g., embracing) is expected upon greeting others. Most reported that, as the pandemic continued, people were less likely to comply with social distancing, especially in summer months, when large gatherings were common.

*“[Brazilians] will instinctively shake hands or hug, even my patients. I saw a few patients one-on-one, keeping distance… But the greeting process was off. For them, it was like, everything is normal. They have no problem shaking hands or giving you a hug… They haven’t stopped that behavior completely.”*

—Mental health provider

### 3.6. Impact on Employment, Financial Security, and Ability to Meet Basic Needs

All KIs reported that a large proportion of Brazilian immigrants work in public-facing service occupations such as housecleaning, construction, and food services. As lockdown orders began, the majority of these services were put on hold and unemployment rose precipitously. KIs described that financial need drove many to continue working during the pandemic, even in unsafe conditions. Although obtaining jobs as “essential workers” during lockdown was not possible for many undocumented workers, some took up “gig” employment with grocery delivery services or rideshare apps. These gig positions and other jobs commonly assumed by Brazilians (e.g., construction workers, restaurant staff) were also described as high-risk, where it was difficult for workers to follow CDC safety guidelines.

*“The [jobs] ceased to exist, but people still needed to pay their bills and had nowhere to turn to. And most people in our community are connected to the workforce through informal networks. They don’t have a job that they can do from home. I see a lot of people working for the gig economy, … working for Uber, for Instacart, doing deliveries. Sometimes this is just not safe.”*

—Social service provider

Most (13/16) participants believed that a significant portion of the population faced unsafe conditions at work that increased the risk of being exposed to COVID-19. KIs also described that members of the community were often unable to take time off from work to quarantine. Another factor that increased potential exposure was the fact that many community members live in close quarters with multiple family households. KIs also noted that living in these conditions was particularly challenging when individuals were awaiting test results or trying to quarantine themselves.

*“I believe that many people that should be avoiding working, are not doing that, especially because so many people have lost their jobs, and those who didn’t are trying to do anything they can to keep their jobs, to keep working, and keep their income… Unfortunately, undocumented immigrants, which comprise most of the Brazilian community here, are not entitled to receive government support.”*

—Social service provider

Half (8/16) of KIs reported that domestic workers, including house cleaners, often did not ask their customers if they had contracted or been exposed to COVID-19 out of fear of being fired. KIs reported that these dynamics were made even more difficult due to language barriers between employers and employees:

*“[Customers] want to have the cleaning done. We have people who got sick going there. We provide training explaining the importance of using gloves, asking the customers if they had any symptoms, if they had any contact. But I can see that some, they were not prepared to do that. And they were concerned to ask the customer and the person says, ‘You don’t need to come anymore.’”*

—Healthcare provider

All KIs also reported that high unemployment led to many community members being unable meet basic needs. At the beginning of the pandemic, food insecurity was a major concern as mass unemployment ensued. However, this was assuaged through rapid mobilization of community organizations, local clinics, and city services in addition to food support structures already in place. While the impact of food insecurity was mitigated by these resources, a third (5/16) of KIs reported that Brazilians had difficulty adjusting to the types of food made available through these programs because they were not common in Brazilian cuisine.

*“Food is the one that they had access to the most because people just mobilized, and churches and food pantries like they just started to appear, like I don’t think food was the main issue.”*

—Political appointee

In addition to food insecurity, 14/16 KIs reported that housing insecurity was a significant concern. Many families were unable to pay rent and KIs believed that the temporary eviction moratorium (which expired 17 October 2020, defaulting to a CDC moratorium in place until 31 December 2020 and extended by the Biden administration until 31 March 2021) [27,28] was the only measure preventing large-scale displacement. At the same time, they reported that many Brazilians do not have legal lease agreements, and therefore were left unprotected, even with the moratorium.

*“Some landlords, they’ve been really pushing [tenants]. We had a situation where the landlord came with a gun… So it was very hard for [tenants], for us to help them calling landlords, we still calling landlords. At this point they were like, ‘Oh, you’re okay. You’re going back to work, so you’re supposed to pay me for the three months that you owe me.’ We had the rental assistance, we got more money from the city to help people but still not enough, we have a huge line, a waiting list.”*

—Social service provider

Unmet childcare needs and the demands of helping children transition to online schooling prevented some from returning to work and further exacerbated financial concerns.

*“If you don’t have childcare and you have a child at home, you cannot go to work. So I think childcare was one thing really important and it affected people even returning to their jobs.”*

—Religious leader

The consequences of financial insecurity and unemployment were not mitigated by the financial support provided by the federal government, since many didn’t receive it. For the Brazilian community, this was largely due to documentation status.

*“Tons of people, they were able to go and get their unemployment money. That doesn’t happen with our community if they don’t have the Social Security to get unemployment assistance. So, how do you pay your rent? How do you keep going? How do you pay your bills?”*

—Healthcare provider

In response to unmet needs, KIs reported that community organizations mobilized to fill gaps left by the federal government by providing various services, including food deliveries, health information, and monetary support, to alleviate the impact of COVID-19 and mitigate the virus’s spread.

*“[Brazilian advocacy] organizations… were able to really help during the pandemic, not only politically, but actually physically, bringing food, giving information, connecting people with the hospitals. That was a very good thing, and I’m proud of the work we did and we are doing.”*

—Political appointee

### 3.7. Impacts on Mental Health and Interpersonal Relationships

All KIs believed that mental health problems, including anxiety and depression, which were already highly prevalent among Brazilian immigrants, grew worse during the pandemic. They attributed this to overlapping factors, such as social isolation, financial insecurity, immigration concerns, and fear of the virus itself.

*“We know that depression and anxiety are two major mental health concerns we have in the community. I think that that just became worse. And stressors became much more evident. From the moment that your ability to survive gets shaken, everything else is put under a different context.”*

—Social service provider

Lack of access to mental health services was reported to be a major issue by 10/16 KIs. They attributed this to people having difficulty finding providers who spoke Portuguese and/or understood Brazilian culture.

*“There’s not a lot of resources. There are healthcare providers, culturally and linguistically competent ones maybe, but this is not easily available for people without documentation, and there’s not a lot – they don’t have enough people to go around to meet the demand.”*

—Healthcare provider

Given this lack of access to mental health services, many (10/16) KIs described that many community members relied on faith-based organizations for emotional support through the pandemic. They reported that various churches conducted online services throughout the stay-at-home advisory period, and that these events had a wide digital following.

*“We were doing our services through the internet, just streaming. Now people are coming back… at least once a week, I am in the church all day for pastoral counseling.... Some people who are going through a hard time will call us for prayers or just talking.”*

—Religious leader

Domestic violence, a concern raised by the community prior to COVID-19 [23], was perceived by 14/16 KIs to have been exacerbated during the pandemic, particularly during the stay-at-home advisory period. Many victims who relied on work to distance themselves from their abusers found themselves in constant contact with them, increasing the level of control abusers had in the household. Survivors who initiated court proceedings for restraining orders prior to or during the pandemic found themselves in a difficult situation as courts closed. Even when courts began reviewing requests through remote appointments, KIs reported that survivors who did not speak English fluently had difficulty navigating the system to request translation services and advocate for their needs.

*“The perpetrator is going to use this opportunity, if you need to stay 24/7 with the person who is controlling you in some way, everything is going to increase. The risk of physical or verbal abuse. Some people, they needed to stop their jobs completely. And that increased the anxiety for that couple... We saw more people asking for help, more people being verbally abused, people asking for ways to relieve what they were feeling. Definitely, we can see more cases in the community during this period.”*

—Social service provider

### 3.8. Community Strengths

KIs also reported that certain Brazilian cultural norms were health-promoting, and as a result, the community was better fortified to respond to the pandemic. For example, hygiene is a highly prioritized aspect of Brazilian society. Best practices including frequent hand washing and showering upon entering the home were already commonplace. Additionally, due to Brazil’s robust vaccination program, some (5/16) KIs believed that Brazilians would likely be willing to take a vaccine when made available.

*“I think Brazilian people are vaccine friendly. Because in Brazil, we don’t have a choice. We get vaccinated 1,000 times and we’re used to it. So, I think they’ll be open to it. We’re known for overmedicating ourselves, so we just love any recourses. I think once we’re here for a while, that’s when you become a little reluctant in certain ways. But I don’t think they’ll be too resistant as far as a vaccine. As long as the doctors show a lot of documentation to go get vaccinated.”*

—Political appointee

Also, many (7/16) KIs described Brazilians as innovative and entrepreneurial, and noted that some community members began new ventures tailored to pandemic-specific needs.

*“Brazilians are very creative. And many start making masks, initially, as donations to hospitals. But also, they start selling the masks. Not just masks but, you know, things to put in your hair and things like that as a way to get some income. I saw a lot of people selling prepared foods. Suddenly, everything that you can imagine in terms of Brazilian cuisine was available.”*

—Social service provider

KIs also described high levels of resilience among the Brazilian community despite hardships induced by the pandemic. They explained that many people routinely experience hardship as a result of their documentation status, socioeconomic status, discrimination, and other factors. As a result of these every-day experiences, they felt that Brazilian immigrants are more adapted to managing adversity and exploring alternative solutions.

*“Undocumented people actually have figured things out and are not as bothered as people with secure status who have never experienced what we’re experiencing now.”*

—Social service provider

Additionally, many in the Brazilian community are used to living far away from family members living in Brazil. KIs noted that this situation, coupled with the nature of Brazilian culture as very social and family-oriented, meant that most Brazilians are accustomed to relying on virtual channels to keep in touch with loved ones. While many communities were navigating virtual communication as a primary platform for the first time, the Brazilian community already had well established networks through social networking sites.

*“People use WhatsApp and FaceTime and everything to connect to their loved ones in Brazil when they’ve been here for 10/20 years without being able to go back. So, I think that was an easy transition; I don’t see any struggle in that.”*

—Health service provider

These networks served a dual purpose of facilitating social adaptation and maintenance of relationships, as well as a tool for rapid information dissemination regarding community resources and health information.

*“I never seen so many people working together. We knew everybody but now it’s like we’re closer. All the stakeholders in the community, the religious leaders, even the city departments. This is the good part of the situation that we could see like so many people working together to help.”*

—Social service provider

## 4. Discussion

While our findings suggest that COVID-19 has been widespread in the Brazilian immigrant community, we also identified that many people did not initially take the pandemic seriously until someone they knew in Brazil had contracted or died from COVID-19. This was pervasive throughout the community, thought to be due to widespread reliance on social media and Brazilian news which downplayed the pandemic. Additionally, we found that fears surrounding immigration status served as an obstacle in mitigation efforts like contact tracing. Immigrants, particularly undocumented immigrants, were perceived to be less likely to answer the phone or report close contacts out of deportation fears. Conversely, it was believed that Brazilians would be readily willing to receive a COVID-19 vaccine, largely accredited to widespread mandatory vaccination programs in Brazil.

Low rates of COVID-19 testing, particularly early in the pandemic, could be attributed to lack of health insurance, concerns about documentation status and use of public services due to the Public Charge rule, language barriers, and cultural norms that minimize the gravity of mildly symptomatic illness. Reluctance towards mask-wearing was an issue tied to the spread of misinformation about the virus. KIs thought that the cultural value of physical touch made community members more resistant to social distancing guidelines. Conversely, they described that hygiene is a strong element of Brazilian culture, meaning that regular handwashing and showers when entering the house were widely practiced. 

Due to the large proportion of Brazilian immigrants that work informal jobs and lack legal documentation status [29], many were ineligible for federal financial support, resulting in significant financial hardship. Among those who remained employed or found new jobs during the pandemic, many worked in settings with high levels of exposure and limited employee protections. With massive unemployment, there were many who were unable to meet basic needs for food and were at risk for housing displacement. Without governmental support, many turned to community organizations, who worked together to address those unmet basic needs. 

KIs reported that anxiety and depression, which were already highly prevalent in the Brazilian immigrant community [23,24], were heightened by the pandemic. Here, we found that pandemic-related experiences such as social distancing, the stay-at-home advisory, loss of employment, and financial insecurity further exacerbated mental health issues. Barriers to obtaining mental health support included a relative lack of mental health providers with the linguistic and cultural proficiency to work with Brazilians and a high proportion of the population lacking health insurance. Furthermore, the majority of KIs in our study believed that the stay-at-home advisory escalated abusive situations between domestic partners, particularly because of this period of increased stress where the abuser and victim were in close proximity for extended time. While increased domestic violence has been observed in other communities during the pandemic [30,31,32], our prior study raises serious concerns that women are not likely to report violence to the police due to fears of deportation, having their children taken away, or a further escalation of violence by their partner [23].

We also identified community strengths that played a defining role in the community’s response to the pandemic. We found that many Brazilian immigrants are highly resilient due to having experienced significant hardships prior to the pandemic, including those associated with racial and ethnic discrimination, low socioeconomic status, and undocumented status. Health-promoting cultural norms regarding hand and home hygiene were key strengths. We also identified that Brazilians were well adapted to the use of digital platforms to communicate with loved ones, because there has been high reliance on these platforms to maintain contact with friends and family living in Brazil. Strong virtual networks allowed for a more facilitated distribution of information, and collaboration between various organizations and partners created a more cohesive response to community needs. Additionally, many Brazilians found innovative ways to supplement their income or find new sources of monetary income through the gig economy. Due to the lack of published research at this time, it is difficult to compare our findings with other studies that assessed responses to the pandemic within immigrant communities.

Before discussing implications, we acknowledge study limitations. Findings from this qualitative study conducted in one geographic location can help to generate hypotheses for future research, but the study was not designed to produce generalizable findings. We also acknowledge the fact that 8/16 KIs had also been interviewed in our prior study of Brazilian immigrant women’s health. Prior participation in interviews may lead to expectations about what the study team was trying to find and subsequent tailoring of responses to fit with these expectations. The fact that KIs had similar beliefs about the impact of the pandemic on Brazilian immigrants increases our confidence that the themes identified were reflective of community experiences. We also engaged in “member checking” of our findings [33]; we presented results to study participants to ensure that our overall findings and resulting recommendations accurately reflected their reports. 

Our findings suggest that the COVID-19 pandemic has significantly increased the need for access to both physical (e.g., COVID-19 testing, treatment) and mental health services among Brazilian immigrants in Greater Boston. Prior studies show that as many as half of undocumented immigrants and a quarter of “lawfully present” immigrants are uninsured [10]. Lack of insurance was compounded by absence of a primary care provider who could be contacted to provide referrals for testing [9], which was required in the early stages of the pandemic. Furthermore, the CARES (Coronavirus Aid, Relief, and Economic Security) Act signed into law on 27 March 2020, did not address the cost of COVID-19 treatment. For those who are underinsured or uninsured, the cost of treatment may be prohibitive and deter people from seeking testing and treatment [9]. This not only increases the risk for the individual, but also increases community spread of the virus. 

In this study, we found that concerns about immigration enforcement as well as anti-immigrant policies such as the “Public Charge Rule” likely discourage people from seeking the services they need [5,10]. We argue that this should be lifted. Beyond medical services, these fears extend to other crucial safety nets for low-income immigrant communities, including emergency services and police protection in the context of domestic violence. Our findings indicate that extensive and active involvement of trusted community stakeholders will be an integral step to successfully implement COVID-19 testing, contact tracing and vaccination efforts. Such efforts must also account for language barriers and cultural factors.

In addition to compounding physical and mental health effects of the pandemic and immigrant-specific barriers to accessing care, we found widespread financial insecurity and inability to meet basic needs for food and housing. While the economic impact of the pandemic has been widespread, low-income immigrant communities have been particularly vulnerable due to ineligibility for federal benefits [9,10]. We found that the CARES Act, for example, was not a significant benefit to Brazilian immigrants, even for those who pay taxes [9]. Inclusion in local, state, and federal aid for all immigrants would likely be an effective step to promoting public health.

Community organizations effectively mobilized to address food insecurity, which are models of collaboration and action that could be replicated, if provided sufficient resources, for other basic needs that have been affected by the pandemic. Additionally, our data point to the need for relief efforts to be cognizant of cultural considerations, such as food pantries offering culturally appropriate foods for their target population. Another important aspect of relief efforts is housing security. Given the recurrent mention of predatory landlord practices in our interviews, tenants’ rights protections and eviction moratoriums would likely be effective measures to keep people in their homes, which is known to promote health [34]. 

## 5. Conclusions

The purpose of this study was to understand how the COVID-19 pandemic impacted the social and emotional wellbeing of Brazilian immigrants in the US. Our findings indicate that the Brazilian community has endured a variety of challenges such as financial insecurity, fears about documentation status, and language barriers, which have left them vulnerable to the impacts of the COVID-19 pandemic. Specifically, our findings suggest that COVID-19 infections, mental health issues, and domestic violence were prevalent. While we noted numerous needs, we also identified several community strengths, including a reliance on strong and extensive social networks and hygiene practices consistent with CDC recommendations. Aside from immediate efforts to alleviate the burden of COVID-19 on immigrant communities, there is a need for additional research to fully understand the impact of COVID-19 on these marginalized populations. Given that our findings indicate a shortage of adequate government support, capturing the number of COVID-19 infections and deaths among immigrant communities will help demonstrate the need for more resources. Since our findings indicate the influence of language barriers and a lack of awareness about health guidance and legal rights, we recommend the study and implementation of interventions that focus on the dissemination of information sources in Portuguese that specifically target misinformation, health campaigns that emphasize the importance of mask-wearing and social distancing, and Know Your Rights training that emphasizes safe work practices and tenants’ rights. It is crucial that this research be conducted in a timely manner in order to stop the spread of COVID-19 and mitigate its devastating impacts on immigrant communities.

## Data Availability

The dataset generated and analyzed during the current study are not publicly available but are available from the corresponding author on reasonable request.

## Appendix A

**Table A1:** Thematic Areas and Questions for Key Informant Interviews.

Thematic Areas	Sample Interview Question
**Access to testing, treatment, and barriers**	What has been Brazilian’s experience with the COVID-19 testing process?
**COVID-19 beliefs, behaviors, and attitudes**	How are people reacting to CDC recommendations to stay at home, practice frequent handwashing, wear masks in public, etc.?
**Impact on occupations**	How has COVID-19 impacted people’s work in the community?
**Impact on food, housing security, and basic necessities**	To the best of your knowledge, how has COVID-19 impacted community access to food and other basic necessities?
**Impact on mental health & relationships**	How has COVID-19 impacted mental health?
**COVID-19 relief experience**	What kind of support are Brazilians receiving? Where from?
**Re-opening the country**	How do you believe people in the Brazilian community will respond to a COVID-19 vaccine?

## Appendix B

**Table A2:** Research Themes & Supporting Quotes.

Theme	Supporting Quotes
**Beliefs and Attitudes about the Pandemic**	***Perceptions about COVID-19 infections: *** “Framingham [city with the largest Brazilian populations in MA] was the city from the metro area that has the highest number of infections... The contamination was in mostly housing units, condominiums, and that’s where the Brazilians are located. Brazilians, the other immigrants, and other low-income residents are the part of the population that was largely impacted” - *Political appointee* ***Beliefs about the severity of the virus:*** “Some people are too optimistic and saying, ‘Oh, this is nothing, it shall pass’ and ‘We’ve been through worse,’ and ‘You’ve been through the desert in crossing the country,’ things like that.” - *Social services provider* ***Factors influencing beliefs and attitudes:*** “Social distancing was challenging because Brazilian people, we can’t live away from each other. We can’t be without our cookouts for too long.” - *Social services provider* “Washing hands is something that is part of our culture. Brazilians are well known because they are very hard workers. They do house cleaning. They are almost perfect regarding cleaning. I don’t see a lot of problem washing hands.” - *Health services provider* “Our president is not taking it seriously. Because of Bolsonaro, I can tell that some Brazilians keep saying ‘Oh, this is just a *gripezinha* [little cold] or this is nothing. We don’t need to wear mask.’ 90% of the Brazilians who are here [in the U.S.] voted for Bolsonaro, so I think it is related in some way.” - *Social services provider* ***Information access and misinformation:*** “Some people would try to make the effort of reading the information in English. The governor would say X, and they would understand C and pass it to someone else as A... So there was a lot of people spreading fear in the community, trying to be the messenger but they were doing it wrong” - *Political appointee* “Since the COVID-19 death rate is about one percent, they say that it’s not that serious because they are young, they have good health… and they do not need to worry about that. And like information that children cannot get COVID-19, for instance, is widespread. Many people tell us that they don’t worry about that and that schools should be reopened soon because children are not affected by the disease.” - *Religious leader*
**Access to and Utilization of COVID-19 Testing and Treatment**	“Even those who are not undocumented, who have some documentation, but are not permanent residents… the Trump Administration expanded the list of public charges; people are afraid of going to the doctor at all.” - *Political appointee* “If you do manage to get tested, then there is this delay in the result...So, ‘I get tested, I’ve been back in the house for five days, if I’m gonna infect anybody, I’ve already infected them, so who cares? And I’m also the breadwinner, so I’m gonna go back to work.’ Until we provide testing that’s immediate, on the spot, and with very quick turnaround for results, we’re not gonna have an effective system of controlling the disease, at least on these CDC models.” - *Social services provider*
**Responses to Mitigation Efforts**	“Well, they do not trust these government services, and that’s why they do not look for support from the government, and contact tracing is something that the government is doing in order to control the pandemic. And I believe if, for instance, someone calls them or reach them at their homes, they will probably not – many of these people will probably be very unwilling to help.” ****- *Political appointee* “... they can only enter the Consulate if they are wearing masks, and they have to clean their hands with alcohol when they enter the building. And we know that many people that go there, they complain about that because they say that it’s not necessary… That’s what we have been seeing in the community.” ****- *Social services provider* “We have been receiving reports of gatherings, even when the crisis was more severe than now. I believe now [July] that the state is reopening their economy and the cases are much less than we had a few months ago, that has probably made people have more gatherings than. We see many pictures on the beaches and parks and restaurants, and I believe the Brazilian community is not different from them, they are having more gatherings now than before.” - *Social services provider*
**Impact on Employment, Financial Security, and Ability to Meet Basic Needs**	***Unemployment:*** “But most of the fields that Brazilian people, that immigrants usually work in, got canceled. Restaurants, salons, cleaning, babysitting… The Hispanic community does a lot more of the ‘essential work’ of janitors and airports and supermarkets and the people that actually continued to work. But the Brazilian ones, their line of work got canceled.” - *Social services provider* “The only people that have continued to work were construction. And a lot of them worked sick and contaminated other people where they worked.” - *Social services provider* ***Impact on food & housing security:*** “The only thing I noticed, sometimes the food that they provide is not cultural foods. Brazilians love rice and beans, and usually they are given different kind of [canned] foods that are not part of the Brazilian diet. It’s a help, definitely it is. But for some, it’s still a problem.” - *Mental health provider* “One thing that happens in the Brazilian community here, is that many people have these informal rent contracts, they do not sign a formal contract, they just rent a room. There are houses where many families live together. And since these are informal contracts, so many people started being forced to leave these houses and had a lot of problems.” - *Social services provider* ***Other basic necessities: *** “A lot of parents, they don’t speak English. A lot of parents don’t know how to help their kids with homework or anything and with the online challenge, it was very hard. I heard a lot of parents saying, ‘I’m getting crazy here, between my own sanity and my kids, and trying to entertain my kids and help my kids at least perform the basics at school,’ and it was very challenging. Sometimes, the kids are left on their own to figure out because of the challenge of the language.” - *Social services provider* ***Lack of support from federal government:*** “Unfortunately, undocumented immigrants, which comprise most of the Brazilian community here, are not entitled to receive government support. For instance, that check that many people received here, and these undocumented immigrants could not receive it. So, these people had to try to keep their income as much as possible and working even against the CDC recommendations.” - *Health services provider* ***Support from community organizations: *** “Pretty much, if they are not—if they did not get the stimulus check—which a lot of them did not—but if they were not getting it, the help of community organizations has been essential, have really been the only way around of getting help because otherwise, there’s really no place that they can go.” - *Political appointee*
**Impacts on Mental Health and Relationships**	***Mental health experiences:*** “Mental health is a big challenge, a long-lasting one. We are seeing it now, we are seeing it ticking. When the state shut down, people held their breath and said, ‘Okay, it’s gonna be one month, two months,’ and it’s not gonna be a short recovery. People are feeling the domino effect of the whole pandemic, not only the health crisis, but again, a financial crisis for people, and a mental health crisis. I think there is a lot of need for mental health support.” - *Mental health provider* “There are not enough resources for the Brazilian people. Another thing is because it’s not the language, it’s the culture. Even though somebody can speak very well English, the culture is a universe away different, for your perception and what you’ve been through and where you come from. So, the cultural incompetence of those professionals, sometimes it’s very hard.” - *Social services provider* ***Domestic violence:*** “The police were the first place that they could file for the emergency restraining order. Usually 10 days after, they have a conference call to decide to extend the restraining order. This was difficult because of the language, more difficult than before. If they get to court, they probably gonna provide an interpreter for the client, but the thing is, how to get there? They don’t know what to do, they don’t know if they call someone that’s gonna be on the other side of the line is gonna understand them.” - *Social services provider*
